# Age- and sex-specific differences in the association of serum osteocalcin and cardiometabolic risk factors in type 2 diabetes

**DOI:** 10.1186/s13098-023-01021-0

**Published:** 2023-03-16

**Authors:** Wei Li, Yan Wang, Jie Dong, Ruiqing Di, Xiaojun Liu, Shengyun Liu

**Affiliations:** 1 Department of Rheumatology, The First Affiliated Hospital of Zhengzhou University, NO. 1, Jianshe East Road, Zhengzhou, 450052 Henan China; 2 Department of Respiratory and Critical Care Medicine, The First Affiliated Hospital of Zhengzhou University, NO. 1, Jianshe East Road, Zhengzhou, 450052 Henan China; 3 Pediatrics Research Institute of Hunan Province, Hunan Children’s Hospital, Changsha, 410000 Hunan China; 4 Department of Nursing, The First Affiliated Hospital of Zhengzhou University, NO. 1, Jianshe East Road, Zhengzhou, 450052 Henan China

**Keywords:** Osteocalcin, Cardiometabolic risk, Metabolic syndrome, Type 2 diabetes

## Abstract

**Background:**

Serum osteocalcin levels are closely related to metabolic syndrome and cardiovascular disease. This study aimed to investigate the relationship between serum osteocalcin levels and cardiometabolic risk factors in patients with type 2 diabetes (T2D) according to age and sex.

**Methods:**

This cross-sectional study included 1500 patients with T2D (991 men and 509 women) aged ≥ 18 years old. The age- and sex-specific disparities in glycemic and lipid control, as well as cardiometabolic risk factors were evaluated.

**Results:**

The levels of serum osteocalcin were significantly higher in women aged > 50 years compared with women aged ≤ 50 years (15.6 ± 6.5 ng/mL vs. 11.3 ± 4.5 ng/mL, p < 0.0001). However, this was lower in men aged > 50 years than men aged ≤ 50 years (12.2 ± 4.2 ng/mL vs. 12.9 ± 4.3 ng/mL, p = 0.0081). We performed correlation analyses of serum osteocalcin and cardiometabolic parameters. Serum osteocalcin concentrations were negative associated with FBG and HbA1c levels in women and men ≤ 50 years old, but not in men aged > 50 years old. Serum osteocalcin were negatively correlated with TG and positively correlated with HDL-C and LDL-C only in men aged ≤ 50 years. In binary logistic regression analysis, serum osteocalcin levels were associated with multiple cardiovascular risk factors, as follows: overweight/obese (odds ratio [OR], 0.944; 95% confidence interval [CI], 0.9–0.991, p = 0.02) in men aged > 50 years; high HbA1C and high FBG in women and men aged ≤ 50 years, but not in men aged > 50 years; after adjustment for confounding factors, high TG (OR, 0.905; 95% CI 0.865–0.947, p < 0.0001), metabolic syndrome (OR, 0.914; 95% CI 0.874–0.956, p < 0.0001), and low high-density lipoprotein cholesterol (OR, 0.933; 95% CI, 0.893–0.975, p = 0.002) were seen in men aged ≤ 50 years only.

**Conclusions:**

Serum osteocalcin level has significant relationships with cardiometabolic risk factors and several age- and sex-related differences in patients with T2D. Decreased serum osteocalcin levels are associated with a worse cardiometabolic risk profile.

## Introduction

Cardiovascular disease (CVD) and type 2 diabetes (T2D) are the two most common cardiometabolic disorders worldwide and are closely linked in the potential of pathophysiology mechanisms [1, 2]. The risk of developing CVD is two-fold higher in subjects with T2D, and CVD is the leading cause of mortality in T2D populations [3]. A cluster of cardiometabolic risk factors, including obesity, dyslipidemia, insulin resistance, and metabolic syndrome (MetS), appear to be potential causal factor for both diseases [4]. According to recent studies, bone is an endocrine organ that can affect multiple physiological processes through the secretion of bone-derived hormones [5]. The complex crosstalk between the bone and other metabolic and cardiovascular tissues has been demonstrated [6].

Osteocalcin, a marker of bone formation, is predominantly produced by osteoblasts. Many studies have demonstrated that osteocalcin, as a bone-derived hormone, is involved in multiple biological processes, including energy metabolism, glucose and lipid homeostasis, cardiovascular health, and stress response [6, 7]. Both carboxylated osteocalcin and undercarboxylated osteocalcin are found in the circulation, the levels of serum total osteocalcin are used as a biomarker of bone turnover [8]. Previous studies suggested that serum osteocalcin concentrations are positively associated with markers of insulin sensitivity and negatively associated with fasting blood glucose (FBG), insulin resistance, body mass index (BMI), fat mass, and MetS [9, 10]. Subjects with T2D or MetS had lower serum osteocalcin levels compared with healthy subjects [11, 12]. Furthermore, osteocalcin levels were decreased in overweight or obese populations [13]. In previous follow up studies, serum osteocalcin was inversely associated with the risk of diabetes, MetS, and mortality due to cardiovascular disease [12, 14]. However, in another study involving elderly or postmenopausal women, no association was found between osteocalcin and glycemic control and cardiometabolic risk [15]. Thus, there are some discrepancies among the results of different studies that need to be explained.

In humans, bone turnover rate varies according to individual variables, among which age and sex are the most important variables determining bone remodeling [16]. Given that the circulating levels of osteocalcin differ by sexes and change with age, the relationship of serum osteocalcin levels with glucose and lipid homeostasis may also differ according to these variables [17]. Furthermore, there are sex differences in cardiometabolic risk profiles [18]. This variability in circulating osteocalcin levels may account for the inconsistent data observed in previous studies regarding the relationships of circulating osteocalcin levels with glucose homeostasis and cardiometabolic risk factors.

The present study aimed to investigate the age- and sex-related differences in the associations of serum osteocalcin concentrations with cardiometabolic risk factors, such as markers of glycemic control, HbA1C, FBG, lipid profiles, obesity, and MetS in patients with T2D.

## Methods

### Study populations

The cross-sectional study populations were recruited from the First Affiliated Hospital of Zhengzhou University between January 2018 and December 2020. Subjects were excluded if they had any known infection, malignant tumors, or were taking medicine that may influence the level of serum osteocalcin. Finally, 1500 patients with T2DM were enrolled. Questionnaires were used to identify the history of medical conditions, family history of disease, CVD history, and other lifestyle factors. BMI was calculated by using the following formula: body weight (kg) divided by height square (m^2^). Subjects were categorized into three groups according to BMI based on the World Health Organization criteria, as follows: normal (BMI < 25 kg/m^2^), overweight (25 ≤ BMI < 30 kg/m^2^), and obese (BMI ≥ 30 kg/m^2^). This study was approved by the Institutional Review Broad of the First Affiliated Hospital of Zhengzhou University.

### Biochemical measurements

Venous blood samples were collected in the morning after fasting overnight. An auto-biochemical analyzer (Roche Diagnostic GmbH) was used to determine the fasting blood glucose (FBG) and serum concentrations of total cholesterol (TC), triglycerides (TG), low-density lipoprotein cholesterol (LDL-C), high-density lipoprotein cholesterol (HDL-C), serum uric acid (UA), fasting plasma insulin, and C-reactive protein (CRP). Glycated hemoglobin (HbA1c) levels were quantified using high-performance liquid chromatography. Serum total osteocalcin level was measured by electrical chemiluminescent immunoassay using a modular E170 analyser (Roche Diagnostics).

### Cardiometabolic risk factor

Systolic blood pressures (SBP) and diastolic blood pressures (DBP) were measured using an automatic blood-pressure meter after sitting for at least 10 min. The average of three measurements was recorded for further analysis. Hypertension was defined as SBP ≥ 130 mmHg and DBP ≥ 85 mmHg or treatment with antihypertensive medication. For cholesterol-related traits, we defined high TC as ≥ 6.21 mmol/L. High TG was defined as ≥ 1.7 mmol/L. High LDL-C was defined as ≥ 4.16 mmol/L. Low HDL-C was defined as < 1.3 mmol/L for women or < 1.03 mmol/L for men. Hyperuricemia was defined as serum uric acid ≥ 420 μmol/L in men and ≥ 360 μmol/L in women. Insulin resistance was estimated based on the homeostasis model assessment of insulin resistance (HOMA-IR) by using the following formula: fasting insulin (mU/L) × FBG (mmol/L)/22.5. Insufficient glycemic control was defined as HbA1c ≥ 7% or FBG ≥ 6.5 mmol/L. An index of overall cardiometabolic risk was calculated by obtaining a summary of the following risk factors: overweight/obesity, hypertension, hyperuricemia, insufficient glycemic control, high TC, high TG, low HDL-C, and high LDL-C. Then, participants were categorized as having low (0–1), medium (2–3), or high (≥ 4) CVD risk status [19, 20].

### Definition of MetS

We used the definition of MetS according to the NCEPATP III criteria [21]. Subjects were classified as MetS when ≥ 3 of the following criteria were met: diabetes; blood pressure ≥ 130/85 mmHg; TG ≥ 1.7 mmol/L; HDL-C < 1.03 mmol/L for men and < 1.30 mmol/L for women; and waist circumference > 102 cm for men and > 88 cm for women.

### Statistical analysis

Continuous variables were expressed as the mean ± SD, whereas categorical variables were represented by percentage. Differences in anthropometric and biochemical variables between participants of each sex were evaluated using a Mann–Whitney U test or the χ^2^-test as appropriate. Spearman correlation analysis was used to evaluate the association between serum osteocalcin level and metabolic variables, such as anthropometric indices and glucose and lipid metabolism-related parameters. Binary logistics regression analysis was used to determine the association of osteocalcin levels with cardiometabolic risk factors according to age and sex after adjusting for various confounders. Statistical analyses were performed using IBM SPSS Statistics for Windows (version 26.0, SPSS Inc, Chicago, Illinois, USA). We considered p < 0.05 to be statistically significant.

## Results

### Characteristics of study population

The anthropometric and biochemical variables related to glucose homeostasis and cardiometabolic risk in both sex groups are shown in Table 1. The average BMI was higher in men than in women (26.7 ± 4.4 kg/m^2^ vs. 25.4 ± 4.1 kg/m^2^, p < 0.0001), and the prevalence of overweight/obesity was significantly higher in men than in women (62.2% vs. 45.6%, p < 0.0001). FBG levels tended to be higher in men than in women (8.5 ± 3.6 mmol/L vs. 8.4 ± 3.3 mmol/L, p = 0.004). However, fasting insulin concentration and HOMA-IR were not significantly different between the two groups. Serum TC, HDL-C, and LDL-C levels were higher in women than in men. The prevalence of MetS was significantly higher in men than in women (69.9% vs. 60.9%, p < 0.0001). However, there are no difference in the use of lipid-lowering drugs or anti-diabetic drugs (mainly insulin) between two groups.

**Table 1:** Study population characteristics according to sex

	Women (n = 509)	Men (n = 991)	P value
Age, years	54.12 (11.167)	46.34 (12.347)	< 0.001
DD, years	6.49 (6.603)	5.05 (6.238)	< 0.001
Smoking, n (%)	39 (7.7%)	376 (37.9%)	< 0.001
Drinking, n (%)	43 (8.4%)	316 (31.9%)	< 0.001
Hypertension, n (%)	225 (44.2%)	425 (42.9%)	0.626
CVD history, n (%)	59 (11.6%)	79 (8.0%)	0.022
BMI, kg/m^2^	25.38 (4.107)	26.77 (4.352)	< 0.001
Obesity, n (%)	232 (45.6%)	616 (62.2%)	< 0.001
CRP, mg/L	6.4202 (29.579)	4.3202 (17.0858)	0.988
HbA1C	8.841 (2.0198)	8.707 (2.2547)	0.577
FBG, mmol/L	8.4039 (3.3211)	8.545 (3.56197)	0.004
Insulin, μU/mL	7.4977 (8.12538)	8.2642 (13.382)	0.516
HOMA-IR	2.72 (3.95)	2.96 (4.06)	0.928
UA, μmol/L	258.68 (60.867)	340.52 (113.668)	< 0.001
eGFR, ml/min/1.73^2^	102.72743 (17.411)	107.5084 (15.968)	< 0.001
TC, mmol/L	4.6619 (1.091)	4.5667 (1.278)	0.019
TG, mmol/L	1.9131 (1.529)	3.0322 (3.151)	< 0.001
HDL-C, mmol/L	1.1762 (0.3219)	1.0122 (0.280)	< 0.001
LDL-C, mmol/L	2.91 (0.944)	2.63 (1.046)	0.002
MetS, n (%)	310 (60.9%)	693 (69.9%)	< 0.001
Serum osteocalcin, ng/mL	14.6646 (6.495)	12.6431 (4.224)	< 0.001
SBP, mmHg	132.54 (19.646)	134.88 (14.145)	0.186
DBP, mmHg	79.82 (10.613)	87.97 (46.707)	< 0.001

*BMI* body mass index, *CRP* C-reactive protein, *CVD* cardiovascular disease, *DBP* diastolic blood pressure, *DD* diabetes duration, *eGFR* estimated glomerular filtration rate, *FBG* fasting plasma glucose, *HbA1c* glycosylated hemoglobin, *HDL-C* high-density lipoprotein cholesterol, *HOMA-IR* homeostasis model assessment of insulin resistance, *LDL-C* low-density lipoprotein cholesterol, *MetS* metabolic syndrome, *SBP* systolic blood pressure, *TC* total cholesterol, *TG* triglyceride, *UA* uric acid

### Serum osteocalcin levels between different age and sex groups

**Fig. 1:**
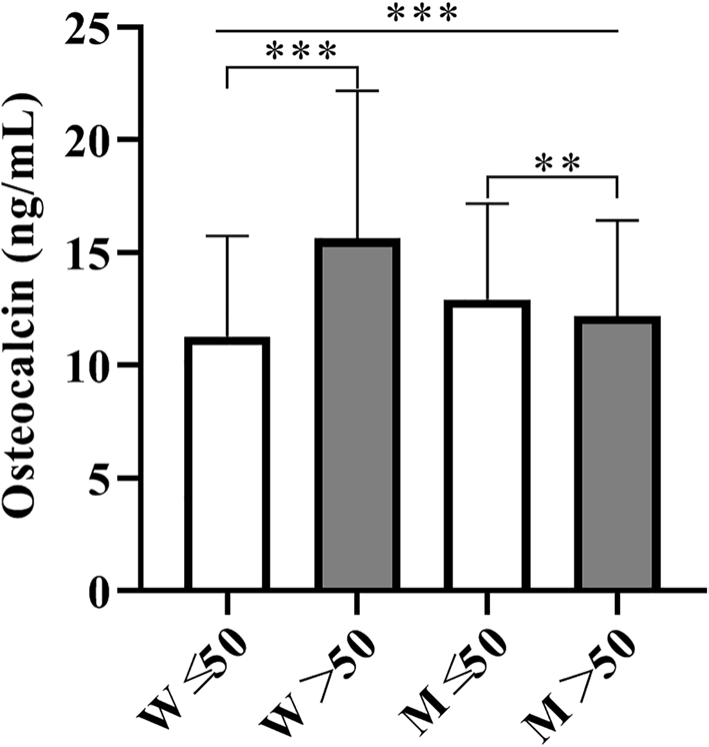
Serum osteocalcin levels according to age, and sex. ^*^p < 0.05, ^**^p < 0.01, ^***^p < 0.0001

Between the sexes, serum osteocalcin levels were significantly higher in women than in men (14.7 ± 6.5 ng/mL vs. 12.6 ± 4.2 ng/mL, p < 0.0001). Considering the trend of serum osteocalcin levels according to sex, we further divided the participants into four groups by age and sex. Serum osteocalcin levels according to age in men and women are shown in Fig. 1. The serum osteocalcin level was significantly higher in women > 50 years of age compared with women ≤ 50 years of age (15.6 ± 6.5 ng/mL vs. 11.3 ± 4.5 ng/mL, p < 0.0001). Interestingly, this was lower in men > 50 years of age than in men ≤ 50 years of age (12.2 ± 4.2 ng/mL vs. 12.9 ± 4.3 ng/mL, p = 0.0081). By contrast, the serum osteocalcin levels were significantly lower in women aged ≤ 50 years than in men aged ≤ 50 years but were higher in women aged > 50 years than in men aged > 50 years (p < 0.0001).

### Relationships of serum osteocalcin levels with other variables

To assess the correlations between serum osteocalcin levels and other variables, spearman correlation analyses were conducted separately for the four groups (Table 2). Serum osteocalcin levels were significantly associated with glucose metabolism parameters, including HbA1c and FBG, except for men aged > 50 years, whereas there was no correlation between serum osteocalcin levels and HOMA-IR in all groups. Serum osteocalcin levels were positively correlated with insulin levels in men aged ≤ 50 years (r = 0.131, p = 0.024) and women aged > 50 years (r = 0.212, p = 0.006). No correlation was found between osteocalcin levels and BMI except in men aged > 50 years (r = ‒0.151, p = 0.002). In addition, the levels of osteocalcin were negatively correlated with TG (r = − 0.191, p < 0.0001) and positively correlated with HDL-C (r = 0.104, p = 0.014) and LDL-C (r = 0.133, p = 0.002) only in men ≤ 50 years of age.

**Table 2:** Correlation of serum osteocalcin levels with other variables according to sex and age group

	Women, ≤ 50		Women, > 50		Men, ≤ 50		Men, > 50	
	r	P	r	P	r	P	r	P
Age, years	0.033	0.7	0.014	0.787	− 0.133	0.001	− 0.085	0.081
BMI, kg/m^2^	0.021	0.812	− 0.103	0.051	− 0.006	0.88	− 0.151	0.002
CRP, mg/L	− 0.01	0.931	− 0.117	0.097	− 0.055	0.312	− 0.032	0.621
HbA1C	− 0.358	< 0.001	− 0.184	0.001	− 0.193	< 0.001	− 0.107	0.03
FBG, mmol/L	− 0.327	< 0.001	− 0.208	< 0.001	− 0.247	< 0.001	− 0.047	0.344
Insulin, μU/mL	0.074	0.525	0.212	0.006	0.131	0.024	0.091	0.21
HOMA-IR	− 0.15	0.195	0.14	0.073	0.073	0.213	0.034	0.642
UA, μmol/L	0.071	0.42	0.03	0.576	− 0.05	0.245	0.037	0.451
eGFR, ml/min/1.73^2^	− 0.172	0.051	− 0.157	0.003	0.003	0.954	− 0.120	0.016
TC, mmol/L	0.017	0.85	− 0.014	0.795	− 0.041	0.331	0.059	0.238
TG, mmol/L	− 0.048	0.585	− 0.091	0.086	− 0.191	< 0.001	− 0.023	0.638
HDL-C, mmol/L	0.027	0.755	0.079	0.134	0.104	0.014	− 0.02	0.687
LDL-C, mmol/L	0.098	0.263	0.022	0.682	0.133	0.002	0.093	0.061
SBP, mmHg	− 0.049	0.564	0.113	0.031	0.022	0.61	− 0.038	0.438
DBP, mmHg	0.027	0.752	0.07	0.182	0.022	0.601	0.019	0.696

*BMI* body mass index, *CRP* C-reactive protein, *DBP* diastolic blood pressure, *eGFR* estimated glomerular filtration rate, *FBG* fasting plasma glucose, *HbA1c* glycosylated hemoglobin, *HDL-C* high-density lipoprotein cholesterol, *HOMA-IR* homeostasis model assessment of insulin resistance, *LDL-C* low-density lipoprotein cholesterol, *SBP* systolic blood pressure, *TC* total cholesterol, *TG* triglyceride, *UA* uric acid

### Association of serum levels of osteocalcin with cardiometabolic risk factors

**Fig. 2:**
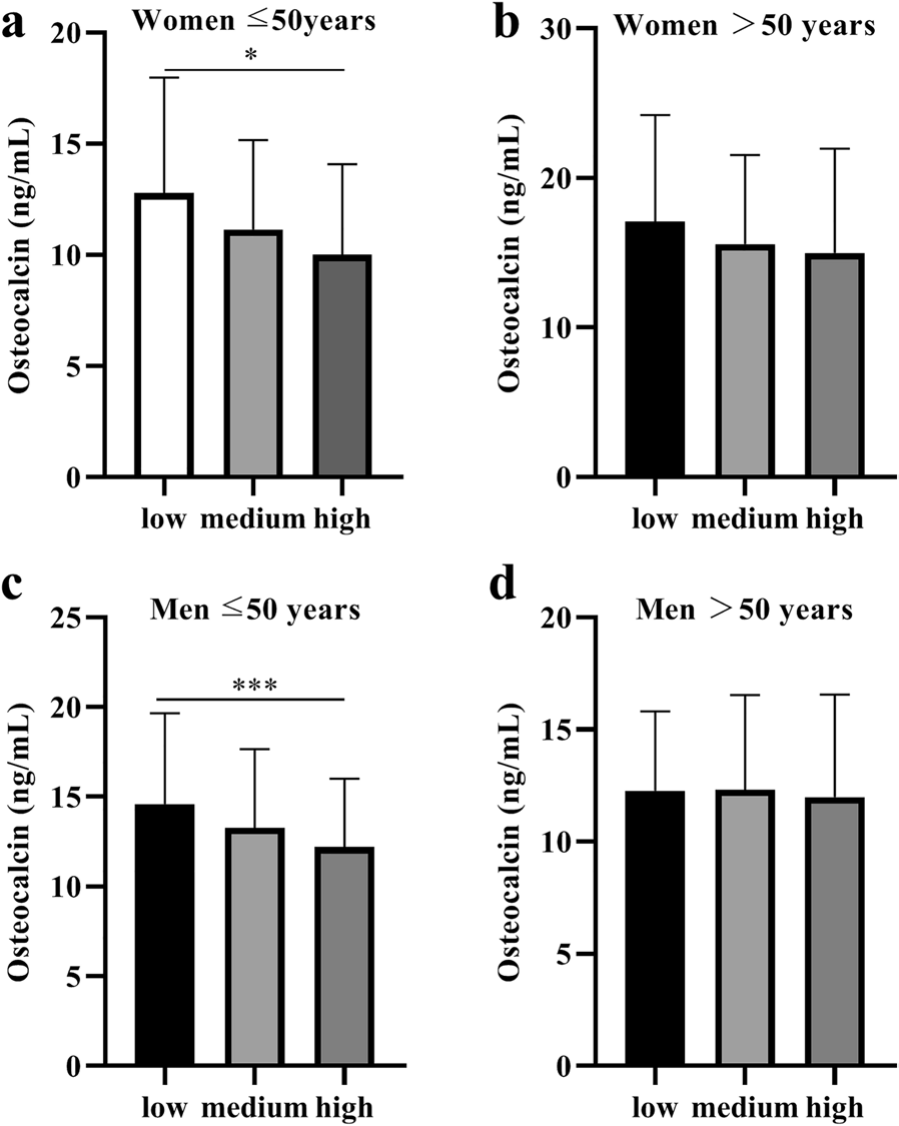
Serum osteocalcin levels in different cardiometabolic risk status according to age and sex. **a** Women ≤ 50 years, **b** Women > 50 years, **c** Men ≤ 50 years, **d** Men > 50 years. ^*^p < 0.05, ^**^p < 0.01, ^***^p < 0.0001

To identify the independent associations of serum osteocalcin concentrations with cardiometabolic risk factors, binary logistics regression analyses were conducted. We adjusted for clinical and biochemical variables, including age, drinking, smoking status, duration of diabetes, CVD history, and BMI. The data demonstrated that serum osteocalcin levels were significantly associated with high HbA1C and high FBG risk in all age and sex groups, except for men aged > 50 years old. The serum osteocalcin levels were also significantly associated with overweight/obesity risk in men aged > 50 years old and inversely correlated with high TG, low HDL-C, and MetS risk in men aged ≤ 50 years old (Table 3). After classifying the participants as having low, medium, or high risk, serum osteocalcin levels were compared according to cardiometabolic risk status. We found that serum osteocalcin levels were significantly lower in those with high cardiometabolic risk status compared with those with low or medium risk status in ≤ 50 years old groups regardless of sex (Fig. 2a, c).

**Table 3:** Association between serum osteocalcin level and cardiometabolic risk factors according to the age and sex

	Women ≤ 50		Women > 50		Men ≤ 50		Men > 50	
	OR (95% CI)	P	OR (95% CI)	P	OR (95% CI)	P	OR (95% CI)	P
Overweight/Obesity	0.991 (0.913, 1.075)	0.827	0.969 (0.937, 1.001)	0.06	0.971 (0.928, 1.015)	0.194	0.944 (0.9, 0.991)	0.02
Hypertension	1.026 (0.933, 1.127)	0.6	1.028 (0.993, 1.063)	0.116	0.995 (0.952, 1.039)	0.806	1.027 (0.979, 1.078)	0.272
High HbA1C	0.833 (0.746, 0.93)	< 0.001	0.944 (0.908, 0.982)	0.004	0.886 (0.845, 0.93)	< 0.001	0.963 (0.913, 1.016)	0.169
High FBG	0.888 (0.809, 0.974)	0.012	0.96 (0.927, 0.993)	0.02	0.893 (0.853, 0.935)	< 0.001	0.989 (0.94, 1.04)	0.66
Hyper UA	1.064 (0.866, 1.308)	0.553	1.023 (0.962, 1.088)	0.466	0.954 (0.895, 1.016)	0.139	1.066 (0.992, 1.146)	0.081
High TC	0.974 (0.81, 1.172)	0.782	0.957 (0.894, 1.025)	0.21	0.943 (0.873, 1.018)	0.134	0.976 (0.851, 1.119)	0.729
High TG	0.983 (0.891, 1.085)	0.74	0.978 (0.945, 1.013)	0.211	0.905 (0.865, 0.947)	< 0.001	1 (0.952, 1.05)	0.992
Low HDL	0.946 (0.86, 1.041)	0.255	0.999 (0.965, 1.033)	0.937	0.933 (0.893, 0.975)	0.002	1.04 (0.99, 1.092)	0.116
High LDL	0.984 (0.832, 1.164)	0.85	0.993 (0.935, 1.055)	0.825	1.015 (0.937, 1.099)	0.722	1.049 (0.928, 1.187)	0.444
MetS	0.976 (0.904, 1.054)	0.542	0.986 (0.954, 1.019)	0.394	0.914 (0.874, 0.956)	< 0.001	1.003 (0.956, 1.052)	0.916

Adjusted for age, drinking, smoking status, duration of diabetes, CVD history, and BMI

*CI* confidence interval, *FBG* fasting plasma glucose, *HbA1c* glycosylated hemoglobin, *HDL-C* high-density lipoprotein cholesterol, *LDL-C* low-density lipoprotein cholesterol, *MetS* metabolic syndrome, *OR* odds ratio, *TC* total cholesterol, *TG* triglyceride, *UA* uric acid

## Discussion

In the present cross-sectional study, which included 1500 patients ≥ 18 years of age with T2D, the serum osteocalcin level was significantly higher in women aged > 50 years compared with women aged ≤ 50 years. However, this was lower in men > 50 years old compared with men ≤ 50 years old. The circulating levels of osteocalcin were negatively associated with FBG and HbA1C in women and men aged ≤ 50 years, positively associated with fasting insulin levels in men aged ≤ 50 years and women aged > 50 old, and negatively related to TG levels only in men aged ≤ 50 years. In the binary logistic regression analysis, we suggested that serum osteocalcin levels were associated with multiple cardiovascular risk factors according to age and sex, including overweight/obesity, high HbA1C, high FBG, high TG, MetS, and LDL-C, after adjusting for potential confounding variables.

Osteocalcin is a marker of bone turnover, and its circulating concentration varies with age according to the rate of bone turnover [22]. In our current study, the serum levels of osteocalcin changed with age in men and women, but there were some differences, as follows. Serum osteocalcin levels were inversely correlated with age in men (r = − 0.142, p < 0.0001) but positively correlated with age in women (r = 0.279, p < 0.0001, data not shown). Furthermore, the average levels of serum osteocalcin were significantly higher in men than in women. By contrast, the serum osteocalcin levels were significantly lower in women ≤ 50 years of age than in men ≤ 50 years of age, but these were higher in women > 50 years of age than in men > 50 years of age. Thus, consistent with previous observations, variables in bone turnover rate lead to age- and sex-specific differences in circulating osteocalcin concentration [15, 23].

Considering the influence of bone metabolism on glucose homeostasis, it could be speculated that serum osteocalcin is associated with glucose metabolism [7, 24]. Some recent studies have suggested that serum osteocalcin levels correlate with glucose metabolism markers, but the results were inconsistent [25, 26]. In the present study, there were significant associations between circulating concentrations of osteocalcin and glucose control in men and women. The data revealed that serum osteocalcin levels are negatively associated with HbA1c and FBG and positively related to serum insulin levels. However, other clinical studies have shown no associations between circulating osteocalcin and markers of glucose metabolism [26, 27]. In most previous studies that explored the association between osteocalcin and glucose metabolism, the investigations were conducted in specific populations, such as older men or postmenopausal women [28]. The differences in bone turnover rates among specific individuals may affect the relation between circulating osteocalcin and glucose homeostasis.

A negative correlation was found between serum osteocalcin levels and BMI and the prevalence of overweight/obese only in men aged > 50 years old, in accordance with previous studies, but these associations disappeared in women aged > 50 years whose bone turnover rate increased and serum osteocalcin levels markedly elevated [17, 29]. Bone formation and resorption occur continuously throughout life, and the bone turnover rate varies according to age and sex. In women, this rate is maintained at a relatively stable level (low level) until menopause. Then, it dramatically increases with increased bone loss [30]. However, the pattern is obviously different in men. Bone turnover rate increases at 20 years of age to reach a peak bone mass and declines slightly after age 50 in men. Thereafter, the serum osteocalcin concentrations remain at a stable level in older men [31]. Thus, the serum osteocalcin level in men do not show dramatic changes at older ages as they do in women at age 50.

Previous study suggested that no relationship exists between osteocalcin and lipid metabolism in T2D [32]. In the present study, the serum osteocalcin levels showed a negative correlation with high TG and low HDL-C risk in men aged ≤ 50 years after adjusting for the other variables. Recent animal study demonstrated that osteocalcin administration ameliorated dyslipidemia and attenuated hepatic steatosis by inhibiting hepatic lipogenesis and promoting fatty-acid β-oxidation [33]. In addition, serum osteocalcin levels showed a negative correlation with MetS risk only in men ≤ 50 years of age. However, these associations disappeared in women and men aged > 50 years. The significant relationship between serum osteocalcin levels and dyslipidemia and MetS risk in men aged ≤ 50 years is a finding that differs from the results of previous studies [28]. The results confirmed the influence of serum osteocalcin on lipid metabolism through a sex-specific approach. Given these discrepant findings, we can speculate that the serum osteocalcin levels in postmenopausal women are more influenced by bone turnover rate than other factors [28]. This finding should be considered when evaluating the association of serum osteocalcin and lipid metabolism. Previous study suggest that a lower proportion of undercarboxylated osteocalcin was associated with better metabolic parameters and lower MetS risk in older man [8]. Further research is need to determine which form of osteocalcin are suitable biomarkers for cardiometabolic risk.

Bone is an endocrine organ that can affect multiple physiological processes through the secretion of hormones [34]. Accumulating evidence supports the idea that osteocalcin has a protective role in cardiometabolic health, and decreased serum osteocalcin contributes to the development of cardiometabolic diseases, partly due to its involvement in glucose and lipid homeostasis [8, 35]. Decreased osteocalcin levels are associated with impaired glucose and lipid metabolism [33, 36]. Furthermore, the present study suggested that lower serum osteocalcin levels were significantly associated with high cardiometabolic risk status in individuals aged ≤ 50 years, regardless of sex. Thus, serum osteocalcin levels were associated with glucose and lipid homeostasis, and cardiometabolic risk should be interpreted according to age and sex. Recent researches have showed complex crosstalks between bone and other metabolic and cardiovascular tissues [6, 37]. However, the underlying pathophysiological mechanism of serum osteocalcin on cardiometabolic health still need to be further explored.

This study has several strengths. We investigated the association between serum osteocalcin and cardiometabolic risk factors in a large number of participants according to age and sex difference. Furthermore, our present study suggested that serum osteocalcin levels were associated with multiple cardiovascular risk factors according to age and sex, including overweight/obesity, high HbA1C, high FBG, high TG, MetS, and LDL-C, after adjusting for potential confounding variables. Remind that the age and sex difference in bone turnover rates should be considered when evaluating the association between circulating osteocalcin levels and cardiometabolic risk.

However, several potential limitations exist in our study. This is a cross-sectional analysis to evaluate associations but not causality, and thus additional studies in larger longitudinal data are recommended to further investigate the age and sex differences in T2DM. The adherence to prescription is objectively documented, but the adherence of the patient to medications is self-reported and might be overestimated. Third, we measured total serum osteocalcin levels only. Previous studies suggest that carboxylation of osteocalcin is important for its biological function.

## Conclusions

Serum osteocalcin level show a significant relationship with cardiometabolic risk factors and several age- and sex-related differences in patients with T2D. Decreased serum osteocalcin levels lead to a worse cardiometabolic risk profile, including hyperglycemia and dyslipidemia, even after accounting for covariates.

## Data Availability

The data reported in this study are available from the corresponding author upon reasonable request.
